# Serial Reduction of an Extremely Large Gastroschisis using Vacuum-Assisted Closure

**DOI:** 10.1055/s-0038-1676045

**Published:** 2018-12-26

**Authors:** Marilyn W. Butler, Julie Fuchs, Matias Bruzoni

**Affiliations:** 1 Division of Pediatric Surgery, Department of Surgery, Oregon Health and Science University, Portland, Oregon, United States; 2 Division of Pediatric Surgery, Department of Surgery, Stanford University, Stanford, California, United States

**Keywords:** gastroschisis, omphalocele, vacuum-assisted closure, VAC, negative-pressure wound therapy

## Abstract

We herein describe a case of serial reduction of an extremely large and complex gastroschisis using vacuum-assisted closure (VAC) therapy in a boy born at 35 ^5/7^ weeks' gestation. A spring-loaded silicone silo was placed at birth. By day of life (DOL) 22, minimal visceral contents had been reduced, and the silo was difficult to maintain due to the size of the fascial defect and loss of abdominal domain. A bespoke VAC dressing was constructed, and biweekly dressing changes allowed gradual reduction of the gastroschisis until the viscera were consolidated. By DOL 50, the viscera were completely reduced, and VAC therapy was discontinued. Feeds were commenced on DOL 57 and increased to goal by DOL 86. The baby was discharged home on DOL 115. We conclude that VAC dressings can be used to aid gradual reduction of an extremely large gastroschisis, particularly in medical fragile infants.

## Introduction

While mortality of gastroschisis in some low- and middle-income countries remains as high as 100%, in developed countries, gastroschisis mortality is an extremely rare occurrence given access to skilled neonatal and surgical care, including ventilator support and total parenteral nutrition. However, complex gastroschisis, whether it is defined by gastrointestinal issues or abdominal wall factors, behaves as a disease process with much worse prognosis. 1

Despite improvements in survival, gastroschisis closure remains challenging, particularly when the fascial defect is large, eviscerated contents are substantial, medical status is precarious, or abdominal domain is lost.

Negative-pressure wound therapy (NPWT), including vacuum-assisted closure (VAC), has been used in children since the 1990s for other challenging wounds to promote the formation of granulation tissue, reduce edema, remove infection, and control fluid losses. 2 3 Previous series have described the application of NPWT for gastroschisis following silo placement after the eviscerated bowel is consolidated. 2 4 5 We herein report the first case where VAC therapy was used to reduce a significantly matted mass of eviscerated bowel in an infant with gastroschisis.

## Case Report

**Fig. 1:**
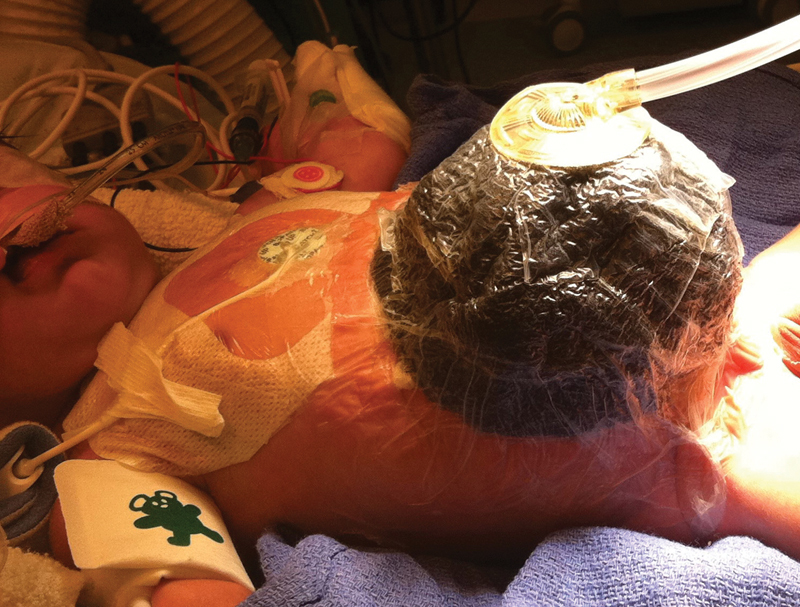
Initial vacuum-assisted closure dressing on day of life 22.

A boy with gastroschisis was born at 35 ^5/7^ weeks' gestation weighing 2.120 kg, to a G2P1 mother through vaginal delivery. Through a defect measuring approximately 4 cm in diameter, a substantial portion of matted abdominal contents was eviscerated, including the stomach, small intestines, and colon. A spring-loaded 5-cm Silicone Silo Bag was placed at birth (Bentec Medical, Woodland, California, United States) and was eventually upsized to a 7.5-cm Silicone Silo Bag. By day of life (DOL) 22, minimal visceral contents had been reduced, and the silo was difficult to maintain due to the large size of the fascial defect and loss of abdominal domain. A bespoke VAC dressing was constructed as follows: Whitefoam and GranuFoam (KCI Medical, San Antonio, Texas, United States) dressings were cut to half their thickness, fashioned in the shape of a cup by sewing strips together, and placed over the eviscerated bowel. Strips of adhesive drapes were used to secure the dressing, circumferentially wrapping the infant. After puncturing the drape, a SensaT.R.A.C. Pad (KCI Medical) was placed over the dressing, connected to a VAC therapy unit, and placed to negative pressure that ranged between 25 and 75 mm Hg ( Fig. 1 ).

**Fig. 2:**
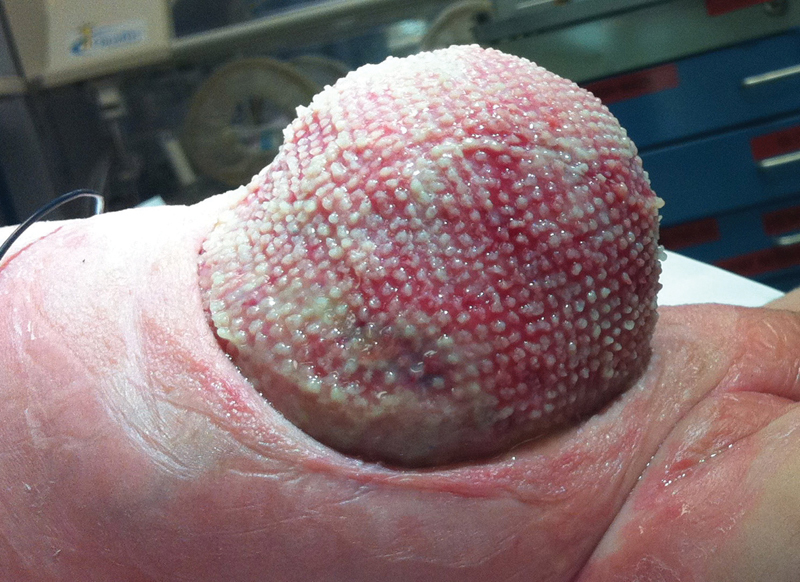
Gastroschisis on day of life 29, 7 days after vacuum-assisted closure dressing was placed, showing consolidation of herniated abdominal contents.

**Fig. 3:**
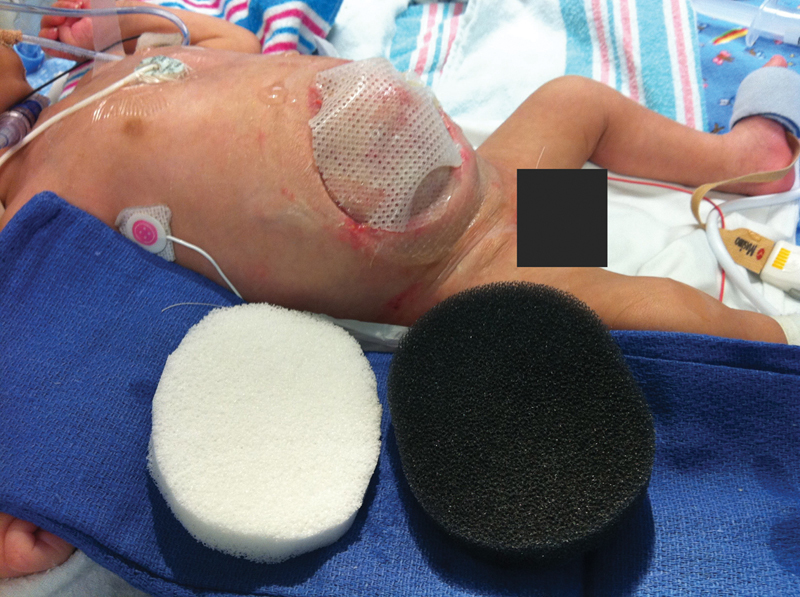
Gastroschisis on day of life 39, 17 days after vacuum-assisted closure dressing was placed, showing dressing materials and nearly complete reduction of herniated abdominal contents.

**Fig. 4:**
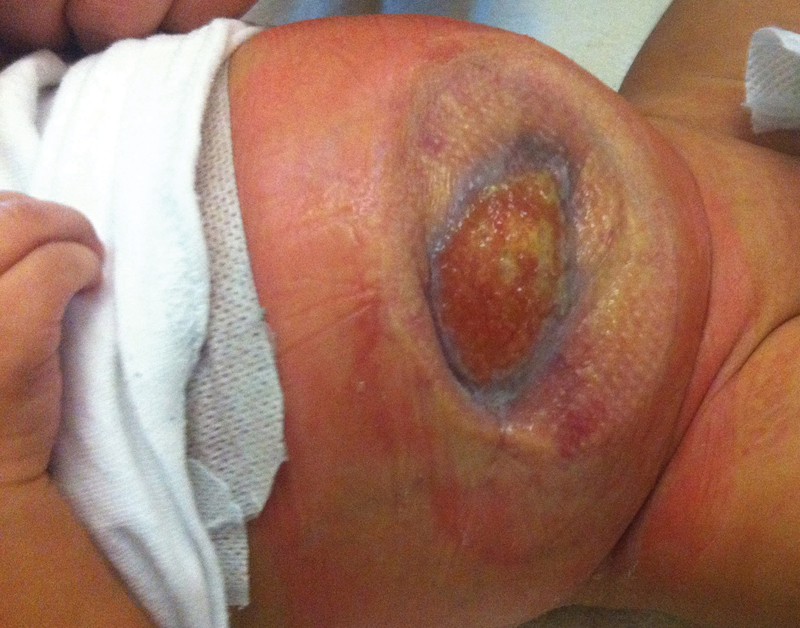
Gastroschisis on day of life 78, showing wound contraction.

Endotracheal intubation was necessary for biweekly dressing changes until the viscera were consolidated ( Fig. 2 ). Then Mepitel (Mölnlycke Health Care, Gothenburg, Sweden) was placed over the viscera prior to Whitefoam and GranuFoam dressings ( Fig. 3 ). By DOL 50, the abdominal contents were completely reduced, and VAC therapy was discontinued. Mepitel was placed until the wound was closed ( Fig. 4 ).

Feeds were commenced on DOL 57 and increased to goal by DOL 86. The baby was discharged home on DOL 115. While initially anemic with cholestasis, by follow-up at 13 months, both had resolved, and his weight was at the 50th percentile for his age. At that point, he was discharged from the clinic and lost to further follow-up. At follow-up clinic visits, the skin had completely epithelialized. An incisional hernia was not noted, although it is presumed that one was present.

## Discussion

Gastroschisis closure has challenged pediatric surgeons for decades. Standard of care for many years entailed immediate primary closure at birth, which resulted in respiratory compromise, abdominal compartment syndrome, bowel ischemia, decreased lower extremity perfusion, anuria, and need for vigorous fluid resuscitation to maintain blood pressure. 6 Occasionally, in challenging cases, silicone sheeting or other plastic materials such as intravenous bags were sewn to the edges of the fascia or the skin to create temporary silos until the viscera could be gradually reduced. 7 8 9 10 Some used pneumatic pressure or wringer clamps to reduce the viscera within the silos. 11 12 With time, however, the fascial defect typically grew larger, and sutures caused the fascia to lose its integrity, making subsequent closure more difficult. In 1995, the introduction of spring-loaded Silicone Silo Bags that could be easily placed at the bedside made delayed closure more convenient, and primary reduction was reserved for newborns whose abdominal contents could be reduced without significant physiological consequences. 13 14 15 In some resource-poor countries, locally sourced female condoms have been used when spring-loaded silicone silos are not available. 16

Despite these advances, in patients with large fascial defects and loss of abdominal domain, use of spring-loaded silos has been unsuccessful in reducing the gastroschisis. At various times, surgeons have used muscle or skin flaps, 17 18 skin grafts, 19 synthetic mesh, 20 biological mesh, 21 amniotic grafts, 22 and pericardial patches 23 to achieve visceral coverage and reduce fluid and heat losses. Skin does not readily cover many of these materials, however, and often mesh needs to be removed 7 17 20 24 or leads to recurrence. 21

More recently, sutureless closure of gastroschisis has incorporated the umbilical cord as a biological dressing for primary repair or delayed closure after silo placement. 25 26 After the cord sloughs, VAC dressings are often used to cover the exposed viscera until the wound is epithelialized. 2 4 5 Until the successful closure in our patient, however, there have been no reports of the use of VAC dressings for primary coverage in gastroschisis that has not yet been reduced. Although our patient did need intubation for the first few dressing changes, once the bowel was consolidated, the VAC dressing could be applied without sedation. Feeds were commenced once the abdomen was closed, and cholestatic liver changes resolved after 13 months.

We do note that our patient's initial management with the silo bag might have made eventual reduction more difficult. Although we generally use the smallest silo bag possible in order not to enlarge the fascial defect, our patient's defect was initially relatively large, and therefore we had no choice than to use a 5-cm silo bag. Upsizing the silo bag made the fascial defect even larger, and in retrospect, were we to encounter the same problem again, we would opt to commence VAC dressings sooner.

We conclude that VAC dressings can be used to aid gradual reduction of extremely large gastroschisis anomalies, particularly in medically fragile infants, without the need for synthetic or biological mesh.

## Notes

New Insights and the Importance for the Pediatric Surgeon When silo closure fails, an extremely large gastroschisis can be gradually reduced with VAC dressings, thereby avoiding the use of synthetic or biological mesh.
